# The Care Home Independent Prescribing Pharmacist Study (CHIPPS)—a non-randomised feasibility study of independent pharmacist prescribing in care homes

**DOI:** 10.1186/s40814-019-0465-y

**Published:** 2019-07-11

**Authors:** Jacqueline Inch, Frances Notman, Christine M. Bond, David P. Alldred, Antony Arthur, Annie Blyth, Amrit Daffu-O’Reilly, Joanna Ford, Carmel M. Hughes, Vivienne Maskrey, Anna Millar, Phyo K. Myint, Fiona M. Poland, Lee Shepstone, Arnold Zermansky, Richard Holland, David Wright, On behalf of the CHIPPS Team

**Affiliations:** 1 Primary Care, Institute of Applied Health Sciences, School of Medicine, Medical Sciences & Nutrition, University of Aberdeen, Foresterhill, Aberdeen, AB25 2ZD Scotland; 2 School of Healthcare, Baines Wing, University of Leeds, Leeds, UK; 3 School of Health Sciences, Faculty of Medicine and Health Sciences, University of East Anglia, Norwich, UK; 4 Norwich Medical School, University of East Anglia, Norwich, UK; 5 Older Peoples Medicine, Norfolk and Norwich University Hospitals NHS Foundation Trust, Norwich, UK; 6 School of Pharmacy, Queen’s University Belfast, Belfast, UK; 7 School of Health Sciences, University of East Anglia, Norwich, UK; 8 School of Healthcare, University of Leeds, Leeds, UK; 9 Leicester Medical School, University of Leicester, Leicester, UK; 10 School of Pharmacy, University of East Anglia, Norwich, UK

## Abstract

**Background:**

Residents in care homes are often very frail, have complex medicine regimens and are at high risk of adverse drug events. It has been recommended that one healthcare professional should assume responsibility for their medicines management. We propose that this could be a pharmacist independent prescriber (PIP). This feasibility study aimed to test and refine the service specification and proposed study processes to inform the design and outcome measures of a definitive randomised controlled trial to examine the clinical and cost effectiveness of PIPs working in care homes compared to usual care. Specific objectives included testing processes for participant identification, recruitment and consent and assessing retention rates; determining suitability of outcome measures and data collection processes from care homes and GP practices to inform selection of a primary outcome measure; assessing service and research acceptability; and testing and refining the service specification.

**Methods:**

Mixed methods (routine data, questionnaires and focus groups/interviews) were used in this non-randomised open feasibility study of a 3-month PIP intervention in care homes for older people. Data were collected at baseline and 3 months. One PIP, trained in service delivery, one GP practice and up to three care homes were recruited at each of four UK locations. For ten eligible residents (≥ 65 years, on at least one regular medication) in each home, the PIP undertook management of medicines, repeat prescription authorisation, referral to other healthcare professionals and staff training. Outcomes (falls, medications, resident’s quality of life and activities of daily living, mental state and adverse events) were described at baseline and follow-up and assessed for inclusion in the main study. Participants’ views post-intervention were captured in audio-recorded focus groups and semi-structured interviews. Transcripts were thematically analysed.

**Results:**

Across the four locations, 44 GP practices and 16 PIPs expressed interest in taking part; all care homes invited agreed to take part. Two thirds of residents approached consented to participate (53/86). Forty residents were recruited (mean age 84 years; 61% (24) were female), and 38 participants remained at 3 months (two died). All GP practices, PIPs and care homes were retained. The number of falls per participating resident was selected as the primary outcome, following assessment of the different outcome measures against predetermined criteria. The chosen secondary outcomes/outcome measures include total falls, drug burden index (DBI), hospitalisations, mortality, activities of daily living (Barthel (proxy)) and quality of life (ED-5Q-5 L (face-to-face and proxy)) and selected items from the STOPP/START guidance that could be assessed without need for clinical judgement. No adverse drug events were reported. The PIP service was generally well received by the majority of stakeholders (care home staff, GPS, residents, relatives and other health care professionals). PIPs reported feeling more confident implementing change following the training but reported challenges accommodating the new service within their existing workload.

**Conclusion:**

Implementing a PIP service in care homes is feasible and acceptable to care home residents, staff and clinicians. Findings have informed refinements to the service specification, PIP training, recruitment to the future RCT and the choice of outcomes and outcome measures. The full RCT with internal pilot started in February 2016 and results are expected to be available in mid late 2020.

**Electronic supplementary material:**

The online version of this article (10.1186/s40814-019-0465-y) contains supplementary material, which is available to authorized users.

## Introduction

### Background

Globally, the population is increasing and ageing. Between 2015 and 2050, the proportion of the world’s population over 60 years will nearly double from 12% to 22% [1]. These changes put pressure on health systems, increasing the need for care. Old age is associated with increasing dependency and vulnerability, declining income and poorer health, and many people require residential care. Whilst globally, the number of beds in nursing and care homes have increased [2]. In the UK, capacity has remained almost static since 2005 [3].

Care home residents are often frail, have multiple morbidities and need high levels of support. Most are prescribed a large number of medicines, rendering them particularly vulnerable to adverse drug reactions and errors [4]. The UK-based Care Homes’ Use of Medicines Study (CHUMS) found several areas of concerns around medicine management in care homes including errors with medication, prescribing, biochemical monitoring and administration [5, 6]. These findings were corroborated in a systematic review which considered interventions to optimise prescribing for older people in care homes [7].

The CHUMS study proposed that one person should adopt overall continuing responsibility for medicines management, working with a lead general practitioner (GP) within each care home. The UK Department of Health Immediate Action Alert [8] arising from CHUMS required primary care organisations, GPs and pharmacy contractors to establish effective joint working strategies to address the identified concerns. However, a recent Cochrane Review [7] suggests that despite the recommendations in the Immediate Action Alert, care remains sub-optimal and more effective frequent medicines management interventions in this setting are required. The Care Quality Commission identified the proper and safe use of medicines as one area of care that requires regular review and which continues to fall below the expected standards [9].

Recent changes in UK legislation, enabling suitably trained pharmacists to prescribe [10], have provided an opportunity for pharmacist independent prescribers (PIPs) to assume the central prescribing-related role in the care home environment proposed by CHUMS. This role could also incorporate medication review and implementation of any identified changes, management of care home repeat prescribing, medicine reconciliation (when care home patients are discharged back from hospital) and support for care home staff in drug ordering and stock control.

Evidence from the UK [11] suggests that practice-based PIPs can prescribe safely and improve patient outcomes for patients with chronic pain compared to both medication review followed by recommendation to the GP and usual GP-led care without any pharmacist review. A similar service model, in which a PIP assumes responsibility for overall management of an individual care home resident’s medicines, monitoring and authorising repeat prescriptions based on individualised pharmaceutical care plans (PCPs), could also improve patient outcomes. The PCPs, as a detailed record of all resident-related medication activities, could be used to communicate prescribing decisions and plans to the care home staff, residents, GP and any other relevant members of the care team.

Despite some successful service-development projects based on this model [12], there is no randomised controlled trial evidence that, in a care home setting, a PIP can improve the clinical outcomes for residents compared to usual care. The feasibility study reported in this paper is part of a programme of work (https://www.uea.ac.uk/chipps/) which follows the Medical Research Council (MRC) guidelines on developing and evaluating a complex intervention [13]. The ultimate aim is to conduct a definitive randomised controlled trial (RCT) to compare the effectiveness and cost effectiveness of a pharmacist independent prescriber managing care home residents’ medication compared to usual GP-led care. Earlier parts of the programme have defined the proposed service and identified logistical and professional barriers and solutions to address them [14] and have devised a training programme for the PIPs to ensure they have the requisite competencies to deliver the service and identified provisional measures of outcome [15].

### Aims and objectives

The aim of this feasibility study was to test and refine the service specification and proposed study processes in order to inform the subsequent definitive cluster randomised controlled trial.

### Specific objectives

The specific objectives were to do the following:

- Test processes for participant identification (pharmacist independent prescribers, GP practices, care homes and care home residents), recruitment and consent, and assess retention rates.
- Determine suitability of outcome measures and data collection processes from care homes and GP practices.
- Assess service and research acceptability to care home residents, pharmacist independent prescribers, GPs and care home staff
- Test and refine the service specification

## Method

### Trial design

This was a single-arm, open feasibility study conducted in care homes for older people. The study was conducted in four locations across the UK, Grampian (Scotland), Belfast (Northern Ireland), Norfolk (England), and Yorkshire (England).

Each location was asked to recruit one eligible general practice, one pharmacist independent prescriber (PIP) and up to three care homes associated with each participating practice. Each GP/PIP/care home triad had a target of recruiting 10 residents.

### Inclusion criteria for participants

#### GP practice

GP practice managing sufficient care home residents to recruit a minimum of 10 residents in up to three care homes. An existing arrangement with a PIP (see below) was preferred.

#### PIPs

Pharmacists registered with the General Pharmaceutical Council/Pharmaceutical Society of Northern Ireland as a pharmacist independent prescriber and following training, provided by the CHIPPS team, demonstrating competence in the service delivery of the proposed intervention

#### Care homes

Care home primarily caring for residents aged 65 years and over, registered with the Care Quality Commission (CQC) (England), Care Inspectorate (Scotland) or Regulation and Quality Improvement Authority (RQIA) (Northern Ireland) as caring for adults aged 65 years and over. Residential, nursing homes and those with both categories of resident were eligible.

#### Residents

Residents under the care of the participating GP practice, prescribed at least one regular medication, aged 65 years and over and permanent resident in the care home, who were able to provide informed consent/assent, or for this to be provided by a nominated representative. Residents were excluded if they were on an end of life care pathway or receiving their care primarily from secondary care.

### Participant identification and recruitment

#### PIPs and GP practices

Between August and September 2016 each location used locally defined strategies (see Additional file 1) and appropriate local networks to obtain expressions of interest (EOI) from GP practices and PIPs, for either participating in the feasibility study or the planned main RCT. Based on earlier qualitative findings [14], final selection prioritised practices that had an established working relationship with a PIP. Details of recruitment are shown in Table 1 below and each location was advised to use locally supported approaches (see Additional file 1).

**Table 1:** Baseline and follow-up recruitment and retention

	Total number invited	Total number of EOIs (%)	Number participating at baseline	Number participating at follow-up (%)
GP Practices	346	44 (11.6)	4	4 (100)
*PIPs	14	6 (42.8)	4	4 (100)
**Care Homes	6	n/a	6	6 (100)
Residents	86	53 (62.2)	40	40 (100) (2 died)

*Numbers based on two areas. In two areas, PIPs were approached sequentially until one agreed

**One care home in each of three areas and three care homes in one area

#### Care homes

The consenting GP practice in each study location invited up to three care homes (see inclusion criteria) served by their practice to note their interest in participating in the study. The care home managers expressing an interest were sent a formal invitation pack by the local researchers (including letter and information sheet). A second or third care home was only contacted if there were insufficient residents in one home.

#### Residents

**Fig. 1:**
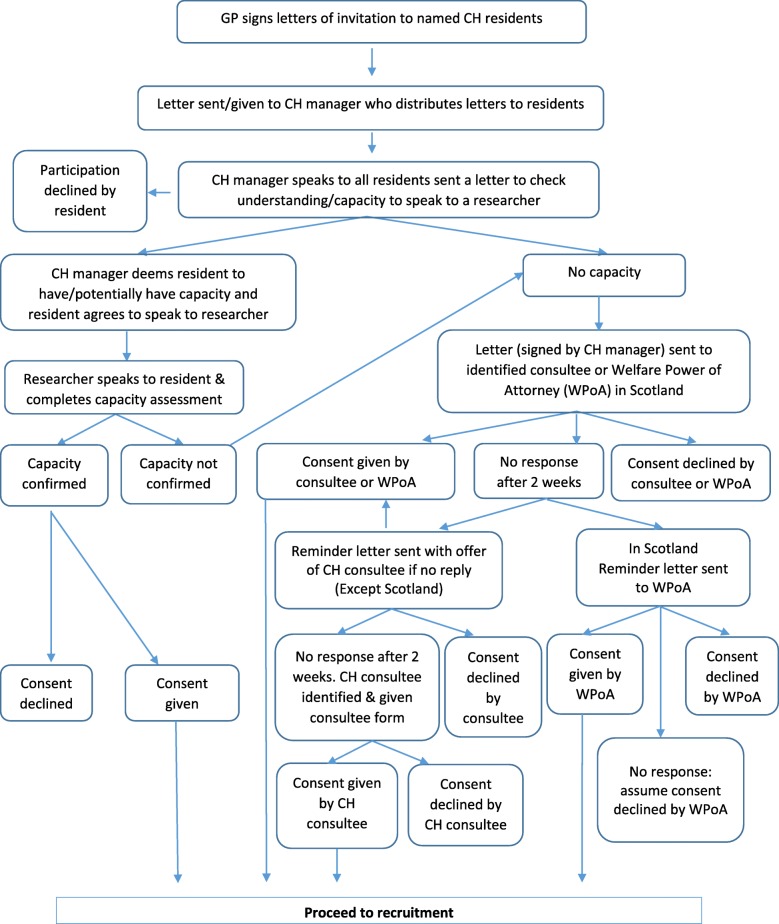
Care home resident recruitment flow chart

Between November 2016 and January 2017, GPs identified eligible care home residents in their care from their computerised lists. An invitation pack (letter from the GP, participant information sheet (spoken version if necessary)) was distributed to each resident via the care home manager. After a minimum of 24 h, the care home manager visited each resident and obtained verbal consent from residents willing to discuss the study further with the study researcher. The local research associate (RA) visited the care home, met with interested residents, assessed their capacity to give consent and took formal signed consent. Where a resident was identified by the care home manager or RA as lacking capacity, a legally appropriate third party (e.g. relative/friend known as consultee (England and Northern Ireland) or welfare power of attorney (WPOA; Scotland)), was contacted by mailed invitation pack. A reminder letter was issued after 2 weeks, if no response had been received. In England and Northern Ireland, if there was no reply from the potential consultee, a consultee from within the care home staff was nominated to provide consent. In Scotland, this is not an approved approach and if there was no reply from the designated WPOA, the resident could not be recruited (see Fig. 1).

GPs were paid for their contribution to patient identification and recruitment and for taking part in the interviews, in line with national guidance (https://www.gov.uk/government/publications/guidance-on-attributing-the-costs-of-health-and-social-care-research).

### Sample size

Since this feasibility study was not designed to assess efficacy, no formal power calculation was conducted. The recruitment target was 10 participants per site (40 in total), in line with recommended practice as judged sufficient to assess feasibility [16].

### The intervention

**Fig. 2:**
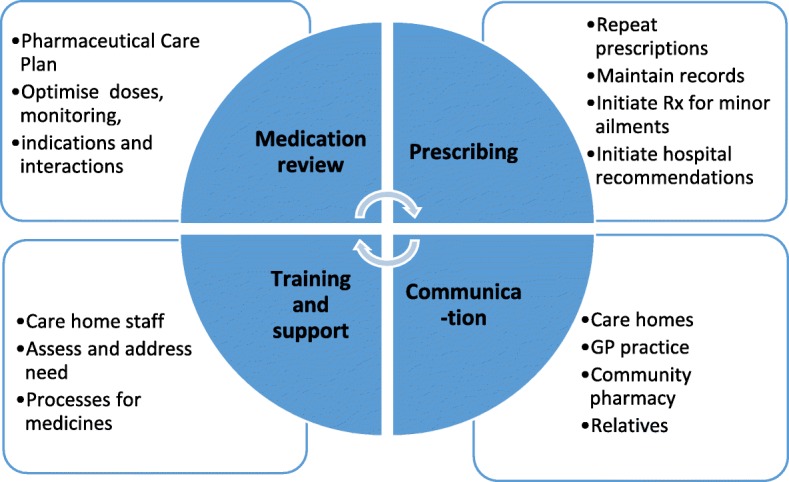
Summary of PIP service

The PIP intervention focused on four key areas including medication review, prescribing, training and support, and communication. The detailed service specification which was developed in earlier parts of the programme is attached in Additional file 2. A summary of the proposed service is shown in Fig. 2 below. The main tasks were reviewing a resident’s medication, developing and implementing a pharmaceutical care plan, prescribing and deprescribing, referral to other health care professionals as agreed with GP, improving communication between GP practice and care home and local community pharmacy, and supporting systematic ordering, prescribing, and administration processes and training for care home and GP staff. As members of the GP team, PIPs all had access to GP records.

The intervention ran for a period of 90 days from February to April 2017. There was an indicative time allocation of 4 h per week per PIP per 10 residents. Pre-intervention, PIPs attended 2 days training (January 2017) which included an overview of the trial design, project delivery, and preparation for the role; the service specification and usage of the PCPs were discussed in depth. This was followed by time to develop relationships with medical practices (for those naive to the practice), care homes and community pharmacists, including performing medication reviews with GPs and observing medication administration by care staff, before final sign-off by clinically qualified professionals independent of the research team. The programme of work which underpinned the development of the training will be reported in full in a separate publication. PCPs were used by the PIP to document each intervention and to communicate any medicines management issues to key stakeholders. A random sample of eight PCPs (two per location) was selected using a random number generator and these were reviewed for appropriateness by one of two grant holders who were specialists in care of the elderly medicine. Assessment was based on professional judgement.

### Estimating the participating proportion of the eligible population

Participants included PIPs, GP practices, care homes and residents. The proportion of pharmacists, general practices, care homes and residents approached who consented to participate was recorded along with the proportion of residents followed-up at 3 months to assess recruitment and retention.

### Suitability of outcomes and outcome measures

Potential outcomes and outcome measures were identified in earlier parts of the programme [15] (see Table 2). All were collected by local researchers at baseline and 3-month follow-up for each participant from records held in the care home, or GP practice, and face to face completion of standard outcome tools with residents and care home staff. To determine their number of medicines suitability for inclusion in the RCT, each of the outcomes/outcome measures was assessed against the following criteria: availability of the data source, potential for bias, potential for missing data, resident centeredness, sensitivity to the intervention (i.e. an indication of change), reliability of data, whether tool was validated, potential for third party completion, ability to blind, time taken to collect per patient and completeness of data. To inform the final selection, performance against these criteria was combined with information on the outcome measure from the literature and general guidance [15]. The minimum criteria for an outcome to be retained in a subsequent RCT were that it should be objective, discriminating, and efficient to collect.

**Table 2:** Outcomes/outcome measures used in the feasibility study

• STOPP/START (medication appropriateness tool) [17]	
• Drug burden index [18]	
• Fall rate per patient as per standard care home safety data collection	
• Adverse drug events as would normally be reported by a care home	
• Mortality	
• Barthel Index (physical functioning) [19]	
• Mini Mental State Examination (MMSE) (Cognitive functioning) [20]	
• Qualidem [21] (dementia specific quality of life)	
• EuroQoL EQ-5D-5 L [2] (Proxy version 1). The proxy (care home staff, key worker) was asked to rate how he or she (i.e. the proxy), would rate the subject’s health (quality of life) [22]	
• EuroQol EQ-5D-5 L face-to-face with resident (quality of life) [22]	

### Assessment of the service and research acceptability and feasibility

#### Participant views

To assess participant views post-intervention, a minimum of one member of care home staff, the care home manager, two residents/relatives and the responsible GP at each location were invited to take part in a face-to-face semi-structured interview. A cross-centre focus group was undertaken with the PIPs, led by study RAs, following a topic guide which included views on the acceptability of the service, and its implementation as well as perceptions of the working relationship with the PIP. All proceedings were digitally recorded and transcribed verbatim.

#### Serious adverse events (ADE)

All admissions to hospital and deaths were recorded as serious adverse events (SAEs) and assessed for causality by a medical doctor on the study management team, using professional judgement.

### Data analysis

Descriptive statistics were used to describe the quantitative outcome measures for baseline and follow-up where appropriate. No statistical comparisons were conducted. Interview and focus group transcripts were thematically analysed using a combined deductive-inductive approach.

## Results

### Recruitment and retention

**Fig. 3:**
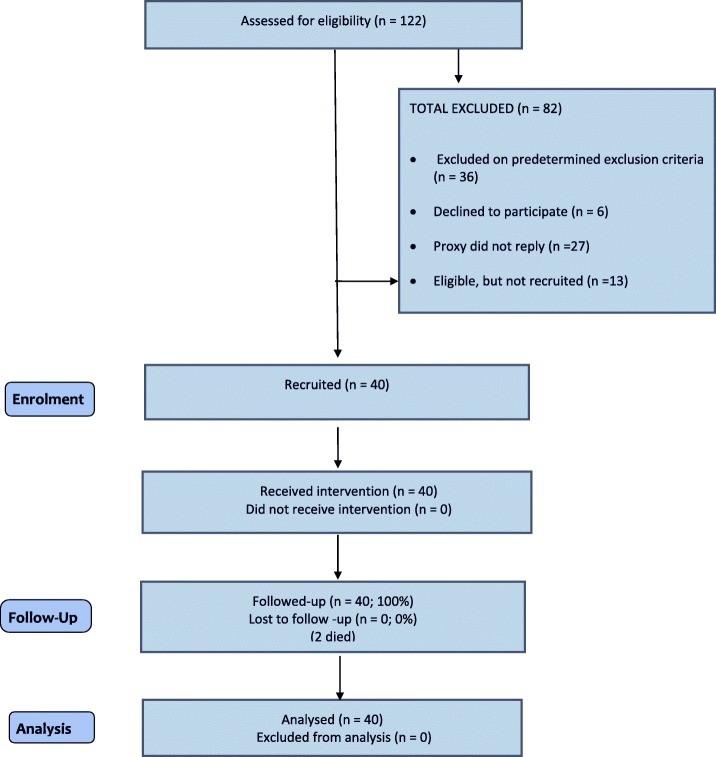
CHIPPS WP5 feasibility study CONSORT Diagram, 20 June 2017

Details of recruitment are shown in Table 1 and Fig. 3 below and varied based on local preferences (see Additional file 1). In one site, invitations were sent to a random sample (100) of all GP practices in their area; in another site, all practices known to be research active were invited (50); whilst in another site, invitations were sent to GP practices known to provide a service to care homes [20]; and in the final site all practices in five clinical commissioning groups (171) were invited and then the PIP approached those expressing interest. Over 11% of GPs invited (11.6% (44/346)) and over 40% of PIPs (42.8% (6/14)) expressed an interest in taking part in either this feasibility study or the subsequent randomised trial. In two areas, potentially eligible PIPs were approached sequentially and the first one agreeing was recruited (see Table 1 and Additional file 1). In the other two areas, a larger number of potentially eligible PIPs were identified and all were invited to take part. The response rate for the PIPs is based on these latter two areas only. All invited care homes agreed to be part of the study. The four recruited PIPs were either employed directly by the practice [1], by the NHS [2] or self-employed [1]. One of the NHS-employed PIPs did not have an existing relationship with the GP.

The GP or PIP assessed 122 residents, identified from GP records, against the study inclusion criteria and excluded 36 (30%). Eighty-six residents were invited to take part, of whom 53 (62%) agreed. In total, 27 (31%) did not reply and six (7%) declined. Of the 53 who agreed, 40 were recruited; the remaining 13 were placed on a reserve list. Over half were female, (24, (60%)), mean age was 84 years (range 67–96 years), and 32 (80%) required nursing care.

In the three-month intervention period, two (5%) residents died. All other residents were retained.

### Suitability of outcome measures

There was data for all standard outcomes (see Table 3) for all or most residents, other than their mental capacity as assessed by the MMSE which could only be completed by 40% and 35% of residents respectively at baseline and follow-up. The direction of change between baseline and follow-up for most outcome measures suggested improvement. The fall rate, the mean Barthel Index, the mean DBI, the number of prescribed medicines and the number of medicines changes as a result of applying the STOPP/START criteria all showed a reduction (i.e. improvement), and the mean MMSE and two Qualidem domain scores (‘care relationship’ and ‘feeling at home’) increased (improved) at follow-up. Whilst the proxy ED-5Q-5 L visual analogue scale (VAS) decreased slightly (i.e. worsened), the self-completed VAS increased (improved). Total Qualidem and six Qualidem domain scores (positive affect, negative affect, restless tense behaviour, positive self-image, social relations and social isolation) all reduced (i.e. deteriorated) in value.

**Table 3:** Data generated by proposed outcome measures at baseline and follow-up

Measure	Baseline	Follow-up
STOPP mean (per patient) (range) SD^#^ Median (IQR)	3.27 (0–7) 1.96 3.0 (2.0–4.25)	2.54 (0–5) 1.24, 2.0 (2.0–3.25)
START mean (per patient) (range) SD^#^ Median (IQR)	2.35 (0–6)1.42 2 (1–3)	2.05 (0–6) 1.55 2.0 (1.0–3.0)
*Falls in past 3 months (total number)	12	10
Falls in past 3 months (number of patients falling)	9/40 (20%)	7/40 (17.5%)
Falls per person (mean (range) SD) Median (IQR)	0.33 (0–3) 0.694 0.0 (0–0)	0.25 (0–2) 0.588 0.0 (0–0)
Barthel mean (range) SD Median (IQR)	6.53 (0–17) 5.50 6.5 (1–9)	6.38 (0–19) 5.51 6.0 (1.0–10.0)
***MMSE mean (range) SD Median (IQR) Number	20.13 (9–30) 7.03 21.0 (14.25–25.5) (*n* = 16)	20.79 (11–29) 5.90 20.5 (15–26) (*n* = 14)
Drug burden index (DBI) score 0 (n (%))	14 (35%)	14 (35%)
**Drug burden index (DBI) *N* score > 0 (*n* (%)) Mean (range) SD Median (IQR)	26 (65%) 1.11 (0.14–3.37) 0.74 1.035 (0.5–1.5)	26 (65%) 0.93 (0.14–3.34) 0.67 0.76 (0.5–1.17
Number of medicines per patient mean (range) SD) Median (IQR) (includes topical preparations)	9.3 (1–26) 5.9 8.5 (6–12)	8.7 (0–31) 6.0 8 (5–10)
Total Qualidem mean, (range) SD, Median, (IQR)	93 (67–130) 15.55 91.0 (79.25–101.5)	77.85 (31–109) 18.32 79.0 (71.0–92.5)
a. Care relationship (mean (range) SD, Median (IQR)	15.33 (10–23) 3.53 14.0 (12.25–18.0)	15.83 (4–21) 5.00 16.0 (13.0–20.0)
b. Positive Affect mean (range) SD Median (IQR)	14.33 (6–24) 5.50 14.5 (9.0–18.75)	12.43 (2–18) 4.33 14.0 (8.5–16.0)
c. Negative Affect mean (range) SD Median (IQR)	8.25 (3–12) 2.25 9.0 (6.0–10.0)	5.35 (0–9) 2.28 5.0 (4.0–7.0)
d. Restless/tense behaviour mean (range) SD Median (IQR)	8.48 (3–12) 2.59 9.0 (6.25–10.0)	5.13 (0–9) 2.41 5.0 (4.0–7.0)
e. Positive self-image mean (range) SD Median (IQR)	7.88 (3–11) 1.88 8.0 (6.0–9.0)	6.23 (0–9) 2.24 6.0 (4.25–8.75)
f. Social Relations mean (range) SD Median (IQR)	12.03 (7–22) 3.68 11.0 (10.0–13.0)	11.13 (1–18) 4.79 10.5 (8.25–15.75)
g. Social Isolation (mean (range) SD Median (IQR)	6.00 (3–11) 1.45 6.0 (5.0–7.0)	4.60 (10–9) 1.99 5.0 (4.0–6.0)
h. Feeling at home mean (range) SD Median (IQR)	8.88 (6–14) 2.22 8.0 (7.0–11.0)	9.68 (1–12) 2.74 11.0 (8.0–12.0)
†EQ 5D-5 L (self) (Mean (range) SD) median (IQR) N	0.449 (−0.281–0.951) 0.335 0.379 (0.279–0.719) *n* = 16	0.572 (0.027–1.000) 0.327 0.585 (0.34–0.77) *n* = 13
†EQ 5D-5 L (proxy) (mean (range) SD) Median (IQR) N	0.434 (−0.027–0.896) 0.229 0.356 (0.267–0.664) *n* = 39	0.406 (−0.019–0.887) 0.236 0.341 (0.206–0.581) *n* = 38
†EQ 5D-5 L Baseline VAS (self) (mean (range) SD) Median (IQR) N	57.5 (0–100) 31.9 67.5 (32.5–75) *n* = 14	62.9 (10–99) 30.2 75 (30–85) *n* = 11
†EQ 5D-5 L Baseline VAS (proxy) (mean (range) SD) Median (IQR) N	51.6 (2–99) 23.9 57.5 (38.75–66.25) *n* = 38	50.2 (2–90) 27.3 50 (30–70) *n* = 37
Adverse drug events related to intervention (ADEs) in past three months	0/40 (0%)	0/40 (0%)

^#^Number of individual medicines which would need to be altered (stopped or started) after researchers with a pharmacy qualification applied STOPPSTART criteria. *NICE (2017) https://www.nice.org.uk/guidance/qs86/resources/falls-in-older-people-pdf-2098911933637, defines a fall as an unintentional or unexpected loss of balance resulting in coming to rest on the floor, the ground or an object below knee level. **Calculation based only on those with any DBI score. †ED5D-5 L index obtained from Devlin et al. [22] on https://euroqol.org, accessed June 2017. ***Average MMSE for the 12 participants who completed the MMSE face to face at both base line and follow-up was 20.5 and 22.1, respectively

Performance of each outcome against the predetermined criteria was combined with data from the literature and discussed by the Programme Management Group. The outcome measure fulfilling most of the criteria was falls per patient, and it was selected as the primary outcome measure for the definitive trial. DBI, hospitalisations, mortality, Barthel (proxy) and ED-5Q-5 L (face-to-face and proxy) and selected items from the STOPP/START guidance also performed reasonably well and were retained as secondary outcomes. Some items from STOPP START were identified as needing clinical judgement and detailed knowledge of the patient’s medical history and these were not recommended for retention. Other measures such as the Qualidem and MMSE were dropped as time-consuming to complete, a better measure was available (to replace the Qualidem), or there was a high potential for bias and missing data/not completed (applied to MMSE) ***(***Additional file 3***)***.

### Adverse events/serious adverse event

Just over 10% of residents (5/40 (12.5%) were admitted to hospital (11 hospital admissions in total, including one resident admitted seven times), and two further residents died (5%). None of the hospital admissions or deaths were judged as being related to the intervention.

### Quality of pharmaceutical care plans

Eight PCPs were reviewed by two doctors. They agreed with all actions documented in six PCPs: two of the care plans were not fully completed and the doctors commented that it was difficult to comment on the changes without the full resident picture.

### Participant views of service and research acceptability

Twenty-eight interviews were conducted across the four sites. Six interviews were completed with each of the care home managers and GPs. Ten interviews were conducted with care home staff, two with residents, three with relatives and one with a dietician. None of those invited declined to take part, although due to lack of capacity the number of residents interviewed was less than the target.

All participants expressed positive views about their experience of the new service. The main themes emerging from analysis were improved patient care, improved patient safety and saving staff time and effort. These are summarised below and illustrated with exemplar quotes identified in italics. Participants are identified only by their professional group due to the small numbers and need to preserve anonymity.

#### Improved patient care

Regular medicine review was reported to lead to improved patient care and quality of life and participants identified utilising the PIPs’ knowledge base and skill set to strengthen the primary care team as beneficial, particularly in vulnerable patients with medically complex multiple morbidities. The key pharmacist skills seen as positively impacting on care provided to residents by supporting, care home staff and GPs were pharmacy knowledge, their ability to prescribe, professionalism, autonomy, ability to provide training and communication. This was summarised by a care home manager: The following quotes from a GP and Care Home Manager illustrate the above summary.

I think you know overall it just had led to better patient care, better medicines management you know for those patients and nursing homes. (GP)

She’s very professional in the fact that you know that she knows what she’s talking about so there’s no question about it. (CH manager)

#### Improved patient safety

Care home managers highlighted the value of the professional support of a pharmacist which had made practice safer including practical aspects of optimising administration routes which required pharmaceutical knowledge. This is illustrated by the following quote:

Swallowing issues, what medicines are suitable for crushing, different formulations … … .. having the pharmacist part of the care home makes it safer again for that reason because GPs will just automatically say oh well crush because we can’t afford to give you liquid. (CH manager)

#### Saving staff time and effort—more efficient

There were several examples where having the PIP on site increased efficiency within the care home and GP practices. In the care home, it was perceived that the PIP was able to have a significant impact on speed of implementation of changes to medication and prescriptions for acute ailments since the PIP had direct access to GP practice computer systems and therefore did not have the same barriers to overcome as care home staff. Care home managers also reported PIPs saved them time as they did not need to verify dosages of medicines, and a dementia nurse highlighted that having a pharmacist who could prescribe was particularly helpful to them. These points are illustrated by the following quotes:

Sometimes I find when you go through GPs it takes much longer if, you know, if you ask them to reduce something, … .then they pass it on. I found with XXX (PIP) after her phone call, it’s implemented straight away, you know, there’s no hanging around, which is good, I like that. (CH manager)

I also know there’s a professional behind me that’s doing something that I don’t have to double check at all. (CH manager)

Well I really welcomed it because I think all the care homes could do with an independent practitioner … … having to wait 48 hours for an urgent prescription and it’s just been horrendous before you came in regards to trying to get what we need from the surgery so having XXX (PIP) here was wonderful. (Nurse)

In addition, the benefits of the PIP having time to communicate with relatives and care home staff were noted and care home managers expressed the view that the PIP had more time than GPs to complete detailed medication reviews for residents:

I think the pharmacist was able to spend more time with us and the resident looking at the medications that they were on, speaking to the staff who knew the residents really well and getting a detailed history which unfortunately we know the GPs haven’t got the time to do that so we thought it was really... … really helpful, yeah. (CH manager)

Time was also freed up for GPs, in particular in reviewing a resident’s medication and being able to work autonomously as a prescriber.

It was very good. XXX (PIP) did most of the work. I had some involvement with looking at the plans and reviewing them and also kind of any bits of advice, but she did most of the work herself and led it herself so I wasn’t hugely involved. (GP)

Care home managers commented that the PIP service did not impact on the day-to-day workload in running the care home and although the PIP needed to discuss resident’s PCPs with the GP, this time was absorbed within the staff workload:

It didn’t really impede on the day to day running of the home, it wasn’t really intrusive to the residents’ day to day lives. (CH deputy manager)

There was some increased workload for the staff because of the time that we needed to give to the pharmacist to discuss the resident in more detail and sort of you know providing the relevant care plans … .. but generally any changes were done at the beginning of the monthly cycle so there wasn’t that much extra work involved. (CH manager)

Finally, it was suggested that the PIP reduced stress levels and improved communication:

Absolutely, if we could have a XXX (PIP) in every single practice, and I know that’s hopefully what’s gonna happen, it would make my job so much easier … . I can see it could make my job a lot less stressful if we had that service right across the board. (CH manager)

#### Potential disadvantages of the service

The main potential disadvantage suggested by care home staff was that the PIP would not be as familiar with the patients as the GP, and GPs expressed concern that they would become less familiar with the residents and the care home staff if they were less involved, as articulated by one GP:

With the lady in question she was saying but the pharmacist doesn’t know her, the pharmacist doesn’t know her history. (CH manager)

because XXX (PIP) is going in and dealing with maybe some of the issues that we would have dealt with in the past, that there’s the potential that you see your patients less and you have less of a close relationship with some patients in the nursing homes so that would be a potential negative going forward … . … … , we may have less contact, but I don’t think there would be any major negative impact from such a scheme. (GP)

### Refinements to service specification

Two of the four PIP pharmacists attended the focus group. Two were unwell on the day and could not attend but were followed-up by individual telephone interview. One interview was recorded and transcribed and the other recorded by notes only due to a failure of the recording equipment. The output from the focus groups and interviews was combined for the analysis.

The PIPs proposed a few minor changes to the service specification. They reported using the service specification to describe the study and discuss the PIP remit in the care home with GPs. They suggested ‘clarification of directions’ for medicines was ‘essential’ and that consultations should be conducted face-to face ‘where possible’, to allow for those relatives who did not live near their relative’s care home. They also commented that delivering the service took more time than had been indicated and suggested some minor simplification to the PCP documentation. The following quote from one PIP, who did not have an existing relationship with the GP practice, suggests that this may lead to challenges in finding time to work together due to other commitments.

… . the engagement from Doctor … .. as the sort of the overall lead GP for that care home, was very disappointing. Initially it started off a little bit more positive in that I did get to shadow him and spend a bit of time with him, but again due to my other roles with the CCG (Clinical Commissioning Group) and also due to his sort of time commitments, we just didn’t seem to be able to catch opportunities to meet and liaise with each other as we ideally should’ve been able to do. (PIP)

## Discussion

### Summary of findings

The findings from this non-randomised study support the feasibility and acceptability of proceeding to the main trial. The processes successfully identified and recruited trial participants (GPs, PIPs, care homes, and residents) and retained them in the study for 3 months. In particular, no problems were identified with the approach to recruiting participants without capacity, including use of consultees and welfare power of attorneys. A range of outcomes/outcome measures was tested and a subset verified as suitable for efficient collection with larger participant numbers. The new service, in which a PIP was introduced into the care home environment was welcomed by all stakeholders. Areas of the service specification that could be changed to enhance the service were identified, but nothing substantive required change.

### Strengths

The success of this non-randomised feasibility study, reported in line with relevant checklist items from recent guidance on randomised pilot studies (CONSORT [23]), reflects the thorough iterative development process. This ensured that the service was tested and the model developed in a way that was acceptable and met stakeholder needs. The feasibility study has allowed the development of detailed Gantt charts detailing timing of recruitment of PIPs, GPs, care homes and residents, randomisation and training of PIPs to fulfil the full programme of work for the definitive RCT. Blinding of RAs at each site when recruiting has also been incorporated into the management of the project to minimise recruitment bias. Selection of the primary outcome/outcome measure for the main trial was based on predetermined evidence-based criteria, using data collected during this feasibility study and from the literature. This removed any bias that might have been introduced by a post hoc subjective process. Finally, our stepwise process to assessing capacity of care home residents to consent ensured all who were able gave informed consent. The process was developed by the team and complies with the implied recommendations for good practice suggested in the literature by DiazOrdaz et al. [24].

### Limitations

Whilst feasibility of the service is confirmed and outcome measures agreed, the challenges of blinding and the willingness of participants to be recruited to a randomised study was not explored within this feasibility study. These uncertainties will be addressed by the internal pilot phase of the definitive RCT. This internal pilot also has defined progression criteria.

A further limitation is a risk of bias. Whilst most outcomes were objective, there was bias when a study team member used professional judgement to assess whether there was a potential causal association between the reported adverse event(s) and the intervention. In the main study, independent GPs will be employed to undertake this assessment following a standard protocol. Similarly, review of the PCPs, conducted by study team members and based on professional judgement, could have been biassed. In the main trial, this process has been standardised with a detailed protocol and reporting templates. The main trial will be advised by continued advice from the independent Programme Steering Committee and a newly convened Data Monitoring and Ethics Committee, and any further changes they suggest to reduce bias will be implemented.

### Generalisability

The findings from four sites, in three devolved nations, demonstrate that the service is feasible in an increasingly divergent UK NHS system. The participant demographics (age, sex, number of prescribed medicines, problems identified) were similar to those of the UK care home population [6], and participation rates were good, suggesting there had not been selective recruitment. The PIPs participating in this study included pharmacists employed by either primary care or the GP practice providing evidence that this service specification is adaptable to either model, although PIPs with a pre-existing relationship with the GP found it easier to arrange meetings. All methods for participant identification, recruitment and follow-up were implemented without any problems being identified and are all scalable. The main trial will use cluster-randomisation by triad (GP, PIP, care home) to avoid contamination. The acceptability of this design to participants remains unknown.

### Feasibility of recruitment process/estimation of the eligible population

Recruitment was smooth at all levels, especially where a more targeted approach to GPs known to work with both a care home and with a PIP in post was adopted. Arrangements for gaining third party consent went smoothly, and these were (post hoc*)* recognised as following published recommendations [24]. Study retention was good with no GP practices, care homes, PIPs or residents lost to follow-up. The level of expressions of interest from eligible GPs confirmed sufficient participants for the main trial and the consent rate of residents has informed the target number of care home patients required to be registered with the participating GP.

### Suitability of outcomes

Following assessment of the outcomes for applicability against the predetermined criteria, falls per patient in the past 6 months was chosen as the primary outcome. Falls have a theoretically informed causal link with the intervention [25], are applicable to a clinically heterogeneous population and are resident centred. The potential for missing data is minimised as the national inspection services (Care Quality Commission/Care Inspectorate/Regulation and Quality Improvement Authority) have mandated and standardised procedures in place for recording falls. Other studies have reported significant reductions in falls of approximately 40% following pharmacist medication reviews [25–27]. The chosen secondary outcomes/outcome measures include total falls, DBI, hospitalisations, mortality, Barthel (proxy) and ED-5Q-5 L (face-to-face and proxy) and selected items from the STOPP/START guidance.

Following accepted guidance [28], all hospitalisations are considered serious adverse events; however, in a population of care home residents, they are relatively frequent and in the feasibility study, their reporting represented a heavy workload, yet none of the events was judged to be associated with the intervention. In the main study, a two-stage SAE reporting procedure will be incorporated into a Safety Management Plan, with only those events with a plausible link to the intervention, as judged by the triad GP, to be reported to the Clinical Trials Unit within 24 h. Further, a proportion of deaths and hospitalisation will be reviewed by an independent GP to ensure unbiased decision-making in assessing whether adverse events are likely to be related to the trial intervention.

In the feasibility study, no other ADEs were reported; therefore, ADEs were discounted as an outcome measure. However, since ADEs are highly significant to patient safety, their reporting will be retained in the RCT as part of the assessment of safety, with systems in place to allow anyone involved in the study to report these.

### Implications for refining the service specification

Care home managers and staff, and GPs all welcomed the intervention and identified benefits of the PIP service. Having the support of an accessible qualified professional who was able to act independently was seen by both GPs and care home staff as improving the quality of care and making processes more efficient. None offered any suggestions for change. The level of detail recorded on the PCP varied between PIPs which highlighted the need for a more consistent approach to completing the PCP which would be addressed during the training of the PIPS prior to the start of the main trial. The PIPs reported that completing the PCP was time-consuming and suggested some changes to ensure the required data could be recorded efficiently whilst avoiding duplication.

## Conclusion

This study has confirmed the acceptability and feasibility of the processes for participant identification, recruitment and informed consent. The PIP service was deemed acceptable to all stakeholders and detailed the particular benefits perceived by GPs and care home staff. Appropriate outcome measures and tools for use in the definitive RCT have been identified, and testing has informed minor refinements to the service specification and protocol for the final phase of this programme of research. An internal pilot phase in the main trial will confirm the feasibility of recruiting and randomising sufficient GP practices, PIPs, care homes and residents, the availability of data for primary outcome at 3 months and that there are no intervention-related safety concerns prior to progression to the main trial. This has started and will complete in mid late 2020.

## Additional files

Additional file 1: Recruitment strategies for GP and PIPs. (DOCX 14 kb)

Additional file 2: Service specification. (DOCX 21 kb)

Additional file 3: Assessment of outcome measures based on data from Cochrane review [7] and experience of feasibility study. (DOCX 16 kb)

## Data Availability

The datasets that support the tables included in this paper are not publicly available due to the terms of the Ethical approval.
