# Supplementary material for: The Care Home Independent Prescribing Pharmacist Study (CHIPPS)—a non-randomised feasibility study of independent pharmacist prescribing in care homes
Source: Pilot Feasibility Stud. 2019 Jul 11;5:89. doi: 10.1186/s40814-019-0465-y (PMC6625047; doi:10.1186/s40814-019-0465-y)
Supplement: Supplementary file 1 — Recruitment strategies for GP and PIPs. (DOCX 14 kb) [file 40814_2019_465_MOESM1_ESM.docx]

***Recruitment Strategies for GP and PIPs***

| **Locations** | **GP Practice** | **PIP** |
| --- | --- | --- |
| **Aberdeen** | Letters of invitation and EOI forms were sent via the Scottish Clinical Research Network co-ordinator to 25 GP practices identified as providing services to Care Homes. Seven GP practices returned EOI forms stating they had a PIP working at their practice. | Seven PIPs linked to the GP practices expressing interest were contacted. The third PIP contacted was happy to take part in the feasibility study and fulfilled all our inclusion criteria. No other PIPs were contacted. |
| **Belfast** | EOI forms were sent out to a random selection of 100 GP practices identified using the Business Services Organisation website. Twelve GP practices expressed an interest to take part but none employed a PIP at that time.  The GP practice that was eventually recruited resulted from a suggestion made by the recruited PIP who had previous experience working with the practice. | EOI were sent to 8 PIPs in Northern Ireland identified using local networks. Four PIPs expressed an interest. The first PIP contacted who could attend the training was recruited |
| **Leeds** | The local Clinical Research Network sent invitations to all 171 GP practices in five CCGS. 11 Expressions of interest were received. The CCG PIP, recruited for CHIPPS, then contacted these GP practices. | PIP was identified by Principal Investigator through local contacts |
| **Norwich** | The Clinical Research Network (CRN) Eastern Primary Care Locality Manager for Norfolk Great Yarmouth and Waveney invited GP practices on the Research Active list. 14 expressed an interest in the study.  Ultimately however the GP practice selected for the feasibility study was approached directly by a PIP based on the PIP’s familiarity with the practice and travel logistics (to ensure that the shortest time possible of the PIP’s 16 hours / month would be used for travel during the intervention). | Norfolk PIPs (6) received invitation e-mails either from the Medicines Management lead for Norfolk CCGs or from the Local Clinical Research Network (LCRN) Pharmacist lead for the East of England.  They were asked to express an interest in the study to the CHIPPS research team. |
