# Supplementary material for: The Care Home Independent Prescribing Pharmacist Study (CHIPPS)—a non-randomised feasibility study of independent pharmacist prescribing in care homes
Source: Pilot Feasibility Stud. 2019 Jul 11;5:89. doi: 10.1186/s40814-019-0465-y (PMC6625047; doi:10.1186/s40814-019-0465-y)
Supplement: Supplementary file 3 — Assessment of outcome measures based on data from Cochrane review [7] and experience of feasibility study. (DOCX 16 kb) [file 40814_2019_465_MOESM3_ESM.docx]

***Assessment of Outcome Measures based on data from Cochrane review (Alldred et al. 2016) and experience of feasibility study***

| **Outcome** | **Data source** | **Potential for bias** | **Potential for missing data** | **Resident centred** | **Sensitivity to intervention** | **Reliability** | **Validated?** | **3rd party completion** | **Ability to blind?** | **Time taken to collect** | **Complet-eness of data** | **Inclusion in RCT** |
| --- | --- | --- | --- | --- | --- | --- | --- | --- | --- | --- | --- | --- |
| **EQ-5D**  (Quality of Life) | Direct interview with resident | High | High | ++ | None of included studies used this measure of QOL. | ++ | ✓ | x | - | 10-15 mins | Poor | Secondary outcome measure |
| **EQ-5D proxy**  (Quality of life) | Care home staff | Low (if completed by same person) | Low | + | None of included studies used this measure of QOL. | + | ✓ | ✓ | - | 3 - 5 mins | Good; 100% | Secondary outcome measure |
| **MMSE**  (cognitive function) | Direct interview with resident | High | High | + | No significant difference reported | ++ | ✓ | x | - | 10-20 mins | Poor | Not included |
| **Barthel Index**  **[proxy]** | Care home staff | Medium | Low | + | No significant difference reported | ++ | ✓ | ✓ | - | 2-10 mins | Good; 100% | Secondary outcome measure |
| **Qualidem**  **[proxy]** | Care home staff | Low | Low | + | Recommend QOL tool for those with dementia | + | Dementia Care Home population only | ✓ | - | 5-10 mins | Good; 100% | Not included |
| ***Falls** | Care home register | Low | Low | + | Three studies found no significant difference, two reported a significant difference | ++ | N/A | ✓ | + | 1 -30 mins | Good | Primary outcome measure |
| **Adverse Drug Events** | Care home record | High | High | + | Verification between medicine & event is difficult to verify. No significant difference reported | - | N/A | ✓ | - | 2-30 mins | None recorded | Monitor for patient safety only |
| **STOPP/**  **START** | GP medical records | High | Medium | - | Significant improvement in two studies | -Concerns about inter-rater reliability- not designed as a research tool | ✓clinical tool only | ✓ | + | 10-20 mins  ) | Good | Secondary outcome measure – reduced list |
| **No. of meds** | GP records | Low | Low | - | Three studies no significant effect  Two studies significant decrease | ++ | N/A | ✓ | + | None | Good | Process measure |
| **Drug Burden Index** | GP record | Low | Low | - | Not previously reported- only relates to anticholinergic medicines | + | ✓ | ✓ | + | 5 mins | Good | Secondary outcome measure |
| **Hospitalisations** | Care home | Low | Low | + | No effect found in three studies  Positive in two | + | N/A | ✓ | - | 5 mins | Good | Secondary outcome measures |
| **Mortality** | Care home | Low | Low | - | No effect reported in five studies | ++ | N/A | ✓ | + | 5 mins | Good | Secondary outcome measure |

*A fall is defined as ‘an event whereby an individual comes to rest on the ground or another lower level with or without loss of consciousness’ (WHO 2007)

N/A not applicable

✓ Meets the criterion

+++, - Extent to which it meets the criterion (fully, partially, does not meet)
